# Supplementary material for: Quantifying inter-annual variability on the space-use of parental Northern Gannets (Morus bassanus) in pursuit of different prey types
Source: PLoS One. 2023 Jul 14;18(7):e0288650. doi: 10.1371/journal.pone.0288650 (PMC10348513; doi:10.1371/journal.pone.0288650)
Supplement: Attachment — Submitted filename: Manuscript_dEntrremont et al.,_NOGA Habitat Suitability ms_PLOSOne.docx [file pone.0288650.s001.docx]

**Quantifying inter-annual variability on the space-use of parental Northern Gannets (*Morus bassanus*) in pursuit of different prey types**

Kyle J.N. d’Entremont^1*^, Isabeau Pratte^2^, Carina Gjerdrum^2^, Sarah N. P. Wong^2^, William A. Montevecchi^1^

^1^Cognitive and Behavioural Ecology Program, Psychology Department, Memorial University of Newfoundland, St. John’s, Newfoundland and Labrador A1C 5S7, Canada

^2^Canadian Wildlife Service, Dartmouth, Nova Scotia B2Y 2N6, Canada

*Corresponding author

Email: [kjde08@mun.ca](mailto:kjde08@mun.ca)

**ABSTRACT**

Spatial planning for marine areas of multi-species conservation concern requires an in-depth assessment of the distribution of predators and their prey in a given region. Northern Gannets *Morus bassanus* are generalist predators that prey upon several different forage fishes depending on their availability. In the western North Atlantic, gannets employ different dive strategies while in pursuit of different prey types, performing deep and prolonged U-shaped dives when foraging on capelin (*Mallotus villosus*), and rapid, shallow, V-shaped dives when foraging on larger pelagic fishes. Therefore, much can be inferred about the distribution and abundance of key forage fishes by assessing the foraging behaviour and space-use of gannets. In this study, we aimed to quantify space-use and to determine areas of suitable foraging habitat for gannets in pursuit of different prey types using habitat suitability models and kernel density utilization distributions. We deployed 25 GPS/Time-depth recorder devices on parental Northern Gannets at Cape St. Mary’s, Newfoundland, Canada from 2019 to 2021. To assess the influence of environmental variables on gannets foraging for different prey types, we built three different habitat suitability models: a U-shaped dive model, and two V-shaped dive models, namely during early and late chick-rearing. Suitable foraging habitat for capelin, deduced by the U-shaped dive model, was defined by coastal and shallow waters with flat relief and sea surface temperatures (SST) of 11-15° C. Suitable habitat for early V-shaped dives was defined by shallow and coastal waters with steep slope and SST of 12-15°C and ~18°C, likely reflecting the variability in environmental preferences of different prey species captured when performing V-shaped dives. Suitable habitat for late V-shaped dives was defined by shallow coastal waters (<100m depth), as well as waters deeper than 200 m, and by SST greater than 16°C. We show that space-use by gannets can vary both within and between years depending on environmental conditions and the prey they are searching for with incidence on the extent of potential interaction with fishing vessels. Further, we suggest regions defined as suitable for U-shaped dives are likely to be the habitat of several species of conservation concern, as these regions are likely to represent consistent capelin spawning habitat.

**Key Words**: Northern Gannet, habitat suitability modelling, GPS telemetry, foraging behaviour, capelin, Atlantic mackerel, Atlantic saury

**INTRODUCTION**

Anthropogenic stressors are negatively impacting marine ecosystems worldwide, particularly in coastal environments where human activity is most concentrated (Halpern et al. 2008). Marine birds are susceptible to harm from many of these stressors, including light pollution (Gjerdrum et al. 2021, Wilhelm et al. 2021), chronic oil pollution (Wiese and Robertson 2004, Robertson et al. 2014), and depleted forage fish populations due to overfishing and climate-induced shifts in distribution, phenology, and regional predictability (Gulka et al. 2020, Montevecchi et al. 2021, Pelletier and Guillemette 2022, d’Entremont et al. 2022a, Montevecchi 2023).

Coastal southeastern Newfoundland (NL) is a region where a high degree of anthropogenic activity and large populations of marine birds overlap. In this region, anthropogenic risks of greatest concern for marine birds include marine traffic (fisheries, shipping, oil transport, and recreational use), bycatch in longlines and gillnets, light pollution, and chronic ship-source oil pollution (Lieske et al. 2019, 2020, Gjerdrum et al. 2021). Climate change and overfishing have also been linked to massive declines in biomass and recruitment success of three key forage fish species in this region: Atlantic mackerel (*Scomber scombrus*, Plourde et al. 2015, Smith et al. 2020), Atlantic herring (*Clupea harengus*, DFO, 2020) and capelin (*Mallotus villosus*, Buren et al. 2019).

The Northern Gannet (*Morus bassanus*) is the largest breeding seabird in the North Atlantic, and is a generalist, opportunistic feeder, that forages on a wide range of fish species depending on its availability, which have changed dramatically within and between years (Montevecchi 2007, Garthe et al. 2011, d’Entremont et al. 2022b). Northern Gannet colonies in the western North Atlantic, including the southernmost colony in the world at Cape St. Mary’s, NL, have exhibited record low productivity in recent years, as well as asymptotic population growth, which have been associated with declines in prey availability due to over-exploitation by humans and climate change (Guillemette et al. 2018, d’Entremont et al. 2022a, Pelletier and Guillemette 2022).

Northern Gannets use different foraging tactics depending on the prey type they are foraging upon. In the western North Atlantic ecosystem, these differences are quantified by the dive profiles, with prolonged, deep, U-shaped dives associated with pursuit and capture of capelin, while shallow, V-shaped dives associated with rapid capture of mackerel, Atlantic saury (*Scomberesox saurus*) and Atlantic herring (Garthe et al. 2000, 2011, 2014, d’Entremont et al. 2022b). Parental Northern Gannets breeding at Cape St. Mary’s, NL, demonstrate distinct shifts in dive profiles, with U-shaped dives falling out of favor to almost exclusively using V-shaped dives as the breeding season progresses (d’Entremont et al. 2022b). Modelling habitat suitability for locations of different dive profiles allows us to delineate key foraging areas and how they might differ based on date and pursuit on differing prey types. These models can also be used as a proxy to identify key areas and environmental drivers for the distribution of these key forage fish (REF).

Our main objectives were to 1) identify key foraging areas for Northern Gannets in southeastern Newfoundland, and 2) determine if Northern Gannet foraging areas differed when using different foraging tactics (*e.g.,* dive profiles). From this assessment, we aim to provide information on how environmental factors may influence the distribution of Northern Gannets and their prey, for a better understanding of the critical areas for parental gannets to ascertain a more effective conservation management in southeastern NL.

**METHODS**

*Study Area and Species*

Cape St. Mary’s Ecological Reserve (46.81°N, 54.18°W) is located at the southwestern tip of the Avalon Peninsula in eastern Newfoundland, Canada (Fig. 1). It lies west of Placentia Bay, east of St. Mary’s Bay, and north of the southeastern Grand Banks. This site is home to 14,598 breeding pairs of Northern Gannets as of 2018, which represents approximately 14% of the Atlantic Canadian population (Chardine et al. 2013, d’Entremont et al. 2022). Since 2010, population size has plateaued at this colony, and productivity has been low, similar to what has been observed at the largest North American colony located in Quebec (Bonaventure Island, d’Entremont et al. 2022a). Average incubation time of gannets in the western North Atlantic is ~44 d (late April to late June) with an average chick-rearing period of ~90 d, ranging from early June to October (Mowbray 2020).

Figure 1. Foraging domain (blue) used in the habitat modelling for parental Northern Gannets breeding at Cape St. Mary’s, Newfoundland, Canada (yellow diamond). Isobaths (grey lines) are at 50 m intervals. Grey dots are all diving locations recorded in 2019, 2020, and 2021. SPM = Saint-Pierre-et-Miquelon.

*GPS Device Deployment*

Ten gannets in 2019, 7 gannets in 2020, and 8 gannets in 2021 were fitted with either battery (n=7) or solar-powered (n=18) Ecotone Uria 300 GPS with Temperature-Depth Recorder (TDR) loggers. Five devices were deployed on both July 18, 2019 and August 21, 2019. Seven devices were deployed on July 18, 2020. Eight devices were deployed on July 15, 2021. Tagged birds had chicks that were ~2-4 weeks old when devices were deployed. Tags weighed 13.5g (dimensions: 36x22x12.5 mm) and were attached to the 4 innermost rectrices just below the uropygial gland with Tesa ® tape and cable ties. Birds were captured using extendable noose poles, weighed with a 5kg Pesola ® spring scale, and equipped with Canadian Wildlife Service aluminum bands on their right legs. Mean mass of tagged gannets across all three years was 3547 g ± 459. GPS devices were < 0.5% of body mass for all individuals and the risk of negative tag effects was minimal (Geen et al. 2019). Tags were set to record the location of each bird every 15 min. Dive depth was recorded every 1s after submersion during diving bouts. An Ecotone base station with a directional antenna was installed ~50 m away from the colony to upload data from devices when birds returned to the colony. Devices were set to only record GPS locations when out of range of the base station to conserve battery life.

*Data Processing*

Dive depth, duration, and bottom time from GPS-tagged gannets were determined for each dive using the package “diveMove” (Luque 2007). Dives that were less than 1 m in depth were removed from further analysis, as these may have been associated with bathing bouts and unlikely to be in pursuit of prey. All dives which had a bottom time ≥ 3 s, and/or total duration > 10 s and a depth > 8 m were designated as “U” dives and all dives which had a bottom time < 3 s, and/or durations < 10 s and depth below 8 m were considered “V” dives (see Garthe et al. 2000). Coordinates of the closest GPS location were assigned to a given dive bout if the latter took place within 30 min of the previous GPS signal, and those locations were thus considered foraging locations. Although U-shaped dives peak during early chick-rearing, V-shaped dives are performed by gannets throughout the chick-rearing period. To account for the potential influence U-shaped dive behaviour might have on the occurrence and distribution of V-shaped dives, we divided V-shaped dives in two categories: early and late V-shaped dive. We used the marked drop in daily proportions of U-dives as the cut-off date to classify early vs late V-shaped dive (Fig. 2; 2019 - 16-Aug, 2020 and 2021 - 10-Aug).

Figure 2. Daily proportion of U- and V-shaped dives performed by Northern Gannets. The black vertical line indicates the cut-off point used to classify V-shaped dives occurring early or late (2019 - 16-Aug, 2020 and 2021 - 10-Aug).

*Dive distributions*

We tested for the independence between year and dive shape using a Chi-square test to determine if there was an association in the distribution of dive shapes among years. The spatial distribution of each dive type was analyzed using the package ‘adehabitatHR’ (Calenge 2006) to estimate 50% and 95% kernel utilization distributions (UDs). To average individuality in movement and ensure individuals were comparable, we used the smooth term *h* = 17 km, which was the mean *h* found across all individuals using the *href* method. Since prey distribution and availability is driven by environmental conditions (Carscadden et al. 2001, Hipfner 2008, Buren et al. 2014, Goyert 2014), we assume the two dive profiles can reflect prey type availability or preference (Elliott et al. 2008). Thus, we built three habitat suitability models for each type of dive (U-, early V-, late V-shaped dives).

*Habitat Suitability Modelling*

For the computation of habitat suitability models we extracted environmental variables within the foraging range of parental gannets determined from GPS tracks. This domain was defined by the farthest eastern and western distances from the colony reached by an individual performing a dive multiplied by 1.1 (451 km), then by drawing an arc until reaching the continental shelf drop in the south, and until reaching the latitude of the northernmost location reached by a gannet (see Fig. 1). The study period spanned from July to October in 2019, 2020 and 2021. In each year and for each dive type, three random locations (hereafter “pseudo-absences”) were generated for each GPS device derived foraging location (hereafter “presence”) (see Aarts et al. 2008, Raine et al. 2020). Each pseudo-absence was randomly assigned a date within the range of the study period each year. To build habitat suitability models for each dive type, we included the following covariates: sea surface temperature (SST, ° C; resolution of 0.01°), depth (GEBCO, m; resolution of 0.004°), slope, distance to colony (km) and longitude. For each presence and pseudo-absence, values for all environmental variables were extracted using the package ‘raster’ (Hijmans and van Etten 2012), and distance from the colony (km) was determined.

For each dive type, we determined which variables to include in our final model by first fitting a logistic regression with a binomial distribution to each covariate independently using generalized additive models (GAM) with the R package ‘mgcv’ (Wood 2019). The binomial response was true location (presence, 1), and the randomized background points (pseudo-absence, 0). We ranked each of the univariate models by their area under the receiving operating curve (AUC) score obtained following 10-fold cross validation to determine univariate model fit. We then followed a step-forward selection adding each variable to our final model following their AUC score order and retaining covariates only if their inclusion decreased Akaike’s information criterion (AIC) of the model while keeping a good model fit (AUC), which was evaluated following 10-fold cross validation. No correlation was found between the covariates considered (Spearman rank correlation absolute rho < 0.4).

For the duration of the three-year tracking period, we predicted the daily probability of occurrence of each dive shape within the domain defined for Northern Gannets breeding at Cape St. Mary’s. A grid was overlaid over the domain extent at a resolution corresponding to the coarsest resolution of our final environmental layers (0.01°, ~1000m for the SST layer). For each day of the study period, we extracted values from the covariates included in our final models at the centroid of each grid square. We predicted the probability of occurrence for U, early and late V-shaped dives using the function *predict.gam* (‘mgcv’ package) for each day of the tracking study, obtaining a daily habitat suitability index (HSI) for each dive type. We averaged the resulting HSI raster layers to generate a final predictive map reflecting the average daily probability of occurrence of U, early and late V-shaped dives, and the error of the spatial prediction was summarized by calculating the pixel-specific standard deviation. Given that the only dynamic variable used was SST, minimal differences were noted when using the model to predict HSI for each of the three study years, thus we presented an overall model prediction by averaging daily prediction obtained over the three years.

**RESULTS**

*Dive distributions*

There was a significant association between the distributions of U- and V-shaped dives among years (*χ^2^* = 122.78, *p* < 0.001). For example, fewer U-shaped dives were performed by gannets in 2019 and 2021 (Table 1). There were fewer V-shaped dives during early chick-rearing and more than expected during late chick-rearing in 2019, but this effect was reversed in 2021. Only in 2020 there were more U-shaped dives than expected, and generally fewer V-shaped dives were observed that year (Table 1).

Table 1. Sample size and tracking period (when dives were recorded) for each year during which Northern Gannets were tracked from the Cape St. Mary’s colony, NL. Number of observed dives of each shape in each year, and number of expected dives of each shape in each year and their residuals following Chi-square test (*χ^2^* = 122.78, *p* < 0.001).

|  |  |  | Observed | | | Expected | | | Residuals | | |
| --- | --- | --- | --- | --- | --- | --- | --- | --- | --- | --- | --- |
| Year | N ind. | Tracking period | U | V-early | V-late | U | V-early | V-late | U | V-early | V-late |
| 2019 | 10 | 19-Jul to 29-Sept | 347 | 1074 | 748 | 419 | 1200 | 551 | -3.5 | -3.6 | 8.4 |
| 2020 | 7 | 18-Jul to 26-Sept | 543 | 1075 | 414 | 392 | 1124 | 516 | 7.6 | -1.5 | -4.5 |
| 2021 | 8 | 15-Jul to 09-Sept | 138 | 797 | 190 | 217 | 622 | 286 | -5.4 | 7 | -5.7 |

The spatial distribution of U-shaped dives performed by Northern Gannets appeared consistent across years, with 50% UD areas centered around the colony but extending slightly further east in 2021 (Fig. 3). Early V-shaped dives had an extended distribution compared to the area used for U-shaped dives, especially in 2021 (Fig. 3). The extent of late V-shaped dives was much wider-ranging than the other two dive types, especially in 2019 and 2020 when foraging gannets travelled west to Saint-Pierre-et-Miquelon and to Fortune Bay.

Figure 3. Spatial distribution of U- (red), early (blue) and late (purple) V-shaped dives performed by Northern Gannet tracked from Cape St. Mary’s (yellow diamond) for each year of the study. Utilization distributions are shown by the solid line (50% UD) and the dashed line (95% UD). Saint-Pierre-et-Miquelon (SPM).

*Model predictions*

Response to the suite of environmental variables included in our models predicted that suitable habitat for late V-shaped dives was more extensive and diffused than for U-shaped and early V-shaped dives (Fig. 4), for which the probability of occurrence was more clustered around the southern coast of the Avalon Peninsula. Although when early V-shaped dives co-occurred with U-shaped dives, the former were more likely to be farther from the colony, over Placentia Bay, and generally southern of the Avalon Peninsula in offshore areas (Fig. 4b). U-shaped dives were strikingly more coastal (Fig. 4a). Overall, the predictive habitat models for each type of dive captured the variability observed in the foraging distribution of Northern Gannets across years, but also highlighted areas of lower density (outside the 50% UD).

Figure 4. Spatial habitat predictions for a) U-shaped dives, b) early and c) late V-shaped dives of Northern Gannets tracked from Cape St. Mary’s (yellow diamond) during three breeding seasons. Average of daily habitat suitability index (HSI) scaled from 0 (unsuitable) to 1 (highly suitable) (a, b, c), and standard deviation of daily HSI for d) U-shaped dives, e) early and f) late V-shaped dives. The spatial resolution of the prediction was set to the lowest resolution of our environmental layers (SST, at 1000m). Isobaths (grey lines) are at 50m intervals. Utilization distributions at 50% for each dive type are overlaid on the top panels for each year represented by different line types (solid - 2019, dashed - 2020, dotted - 2021). Saint-Pierre-et-Miquelon (SPM).

All models retained all the covariates tested (distance to the colony, longitude, bathymetry, slope, and SST) as their addition helped reduce AIC values, while maintaining good model fit (i.e., AUC) (Table 2). For U-shaped dives, the model explained 77.3% (adjusted R^2^ = 0.79) of the total deviance with an AUC of 0.98. The model for early V-shaped dives explained 65.7% (adjusted R^2^ = 0.66) of the total deviance with an AUC of 0.96, while the model for late V-shaped dives explained 52.1% (adjusted R^2^ = 0.53) of the total deviance with an AUC score of 0.93.

Table 2. Model selection following step-forward addition of covariates based on the univariate models AUC scores (not shown). Multivariate models AUC and AICc scores were obtained following 10-fold cross-validation. Akaike model weights (*w*) were also calculated to further inform model selection. Final models were selected based on the lowest AICc.

| **U-shaped** | **AUC** | **AICc** | **▲AICc** | ***w*** |
| --- | --- | --- | --- | --- |
| s(dist2col) | 0.941 | 1719.9 | 862.3 | 0 |
| s(dist2col) + s(depth) | 0.976 | 1012.8 | 155.23 | 0 |
| s(dist2col) + s(depth) + s(longitude) | 0.982 | 901.41 | 43.83 | 0 |
| s(dist2col) + s(depth) + s(longitude) + s(SST) | 0.982 | 870.82 | 13.24 | 0.001 |
| s(dist2col) + s(depth) + s(longitude) + s(SST) + s(slope) | 0.981 | 857.58 | 0 | 0.999 |
| **V-shaped early** | **AUC** | **AICc** | **▲AICc** | ***w*** |
| s(dist2col) | 0.932 | 5357.7 | 1436.6 | 0 |
| s(dist2col) + s(longitude) | 0.956 | 4745.2 | 833.11 | 0 |
| s(dist2col) + s(longitude) + s(depth) | 0.969 | 4094.3 | 173.21 | 0 |
| s(dist2col) + s(longitude) + s(depth) + s(slope) | 0.97 | 4085.9 | 164.74 | 0 |
| s(dist2col) + s(longitude) + s(depth) + s(slope) + s(SST) | 0.975 | 3921.1 | 0 | 1 |
| **V-shaped late** | **AUC** | **AICc** | **▲AICc** | ***w*** |
| s(dist2col) | 0.867 | 3503 | 848.78 | 0 |
| s(dist2col) + s(longitude) | 0.899 | 2929.1 | 274.89 | 0 |
| s(dist2col) + s(longitude) + s(depth) | 0.915 | 2794.1 | 139.87 | 0 |
| s(dist2col) + s(longitude) + s(depth) + s(slope) | 0.914 | 2789.1 | 134.87 | 0 |
| s(dist2col) + s(longitude) + s(depth) + s(slope) + s(SST) | 0.929 | 2654.2 | 0 | 1 |

The partial response curves from the GAM models indicate that the probability of occurrence of U-shaped dives decreased strongly beyond ~200 km from the colony, while probability of occurrence of V-shaped dives during both early and late chick-rearing decreased beyond 150 km (Fig. 5). This decline was, however, of a smaller scale, especially for V-shaped dives during late chick-rearing (Fig. 5c). U-shaped and late V-shaped dives were more likely to occur either west (56°W) or east (52°W) of the colony (54.2°W; Fig. 5a, c). Probability of occurrence of U-shaped and early V-shaped dives increased slightly when foraging at shallower depths. This response was different for late V-shaped dives, for which the response to depth was positive both for deeper (ca < 200 m) and shallower waters (ca > 100 m). The response to slope was marginal, with few data points falling over slopes greater than 8°, but also very different among dive types. Overall, late V-shaped dives show a negative response to very flat bottom relief, while U-shaped dive occurrences positively respond to flatter sea bottom (0-5°slope, Fig. 5a), although this response was marginal. U-shaped and early V-shaped dives show similar response in occurrence to SST with slight positive response between 12 °C and 15 °C (Fig. 5a, b). The response of late V-shaped dive to SST was negative when temperatures were below 12°C, and positive above 16 °C (Fig. 5c).

Figure 5. Model variables for estimated probability of occurrence of a) U-shaped dives and b) early and c) late V-shaped dives by Northern Gannets tracked during three breeding seasons (2019-2021) at the colony of Cape St. Mary’s, Newfoundland. The y-axis represents the function of each predictor variable with the effective degrees of freedom (edf) of the smooth term in brackets. 0 on the y-axis corresponds to absence of an effect of the predictor variable on the estimated probability of occurrence. Environmental variables included in the GAMs were distance to colony (Dist2col; km), longitude (°), bathymetry (Bathy; m), slope (°) and daily sea surface temperature (SST; °C); the y-axis scale varies to emphasise model fit. The confidence intervals (dashed lines) are shown at 1 standard error above and below the smoothed estimate.

**DISCUSSION**

This is the first study to quantify the associations between the foraging of the Northern Gannet on different prey types (*e.g.*, U-shaped dives for capelin; V-shaped dives for mackerel, saury, etc.) and environmental variables in the western North Atlantic using habitat suitability models.

We found the number of U-shaped dives was greater than expected in 2020. Increased diving effort is often indicative of decreased prey availability (Grémillet et al. 2008, Regular et al. 2014, Montevecchi et al. 2019), suggesting increased foraging effort on capelin was required in 2020, perhaps because other prey (i.e., mackerel, saury) were not yet available, or that capelin availability may have been generally low. This is consistent with the fact that capelin availability in Newfoundland waters was considered to be lower than usual in 2020 (d’Entremont et al. 2022b, Lescure 2021, G. Davoren pers. comm.). The number of V-shaped dives was greater than expected in both 2019 and 2021, suggesting that there may have been increased effort associated with lower availability of mackerel and saury in these years (d’Entremont et al., 2022a).

*Habitat Suitability in Pursuit of Varying Prey Types*

The U-shaped dive model, associated to foraging on capelin, suggested that suitable habitat for capelin was constrained near the coast, in shallow waters with flat bottom relief, with SST ranging between ~11-15 °C. Plus, the probability of occurrence dropped of as SST increased above the 15 °C mark, which is consistent with the tendency for capelin to only spawn in waters below 12 °C (Davoren 2013, Crook et al. 2017). Moreover, the decreasing probability of occurrence of U-shaped dives as seafloor slope increases seems to be consistent with the typical spawning site characteristics of capelin in coastal Newfoundland, like flat bottom relief and/or depressions on the seafloor confined by gently sloping marine trenches (Penton and Davoren 2012). This further strengthens previous assertions that U-shaped dives by Northern Gannets in the western North Atlantic are primarily associated with foraging on spawning capelin (Garthe et al. 2000, Garthe et al. 2014, d’Entremont et al. 2022b). Thus, as U-shaped dives tend to be specific to capelin in this ecosystem, these habitat suitability models could be considered as a proxy for suitable habitat for capelin. More importantly, the highly suitable areas for capelin within this study are also believed to be regions of critical concern as many aquatic (e.g. Atlantic cod *Gadus morhua*) and avian (e.g. Atlantic Puffins *Fratercula arctica*, Black-legged Kittiwakes *Rissa tridactyla*, Common Murres *Uria aalge*) predators heavily rely on this prey (Piatt 1990, Carscadden et al. 2002, Link et al. 2009).

The early V-shaped dive model suggested that gannets foraging during early chick-rearing are using areas within ~150 km of the colony, in shallow waters with steep slopes, and constrained to SST ranging 11-15 °C or above 18°C. Thus, the effect of SST on early V-shaped dive occurrence is possibly linked with the water temperature preferences of other prey species rather than capelin; indeed, mackerel prefer temperatures ranging 9-13 °C while saury tend to inhabit in warmer waters, around 18 °C (Dudley et al. 1985, Olafsdottir et al. 2019). Therefore, it is plausible that early V-shaped dives can be used when foraging for mackerel and saury as they start to migrate into the region in late July (Leim and Scott 1966, Moores et al. 1985). In the same line, this SST range also includes the thermal preference for adult herring (8-12 °C, Brennan et al. 2016), meaning that V-shaped dives may also indicate the foraging on herring. As V-shaped dives were associated with steep slopes, it is unlikely that gannets were foraging for spawning capelin, which inhabit in more flat bottom relief areas (Davoren 2013, Crook et al. 2017). However, it is possible that some V-shaped dives were in pursuit of small aggregations of capelin and/or capelin migrating to deeper waters away from their spawning grounds (Carscadden et al. 2013).

The late V-shaped dive model revealed that key habitat during late chick-rearing covered a broader geographic range within the study region, that was characterized by relatively shallow waters, steeper bottom relief and SST above 16° C. Later in the season, as SST increases and capelin abundance decreases, gannets forage on other prey types (d’Entremont et al. 2022b), such as saury and mackerel which prefer warmer waters of ~18 °C (Dudley et al. 1985, Olafsdottir et al. 2019).

Overall, the predictions obtained from the models built for each dive type captured the variability observed in the foraging distribution of Northern Gannets across years, and highlighted areas of lower density (outside the 50% UD). For example, this might suggest that the west and inner coast of Placentia Bay represents more favorable habitat for gannets performing U-shaped and early V-shaped dives. Considering the inter-annual variability in space use, such an area might be used in some years, and not others. Finer scale environmental predictors, better knowledge of prey distribution and behaviour, and other factors could refine models, especially for V-shaped dives during late chick-rearing, for which the model only explained 52.1% of the total deviance. V-shaped dives are performed for more than one prey type that could have different habitat preferences, which makes more difficult to capture in a single model. Nonetheless, the results from our models built with different dive types are mirroring what we know about the tolerance of key forage fishes to sea water temperature, and consequently, the behavioural adaptation of Northern Gannets (Montevecchi et al. 2009, 2021).

*Risk Associated with Anthropogenic Activity and Climate Change*

As space-use by gannets ranging from Cape St. Mary’s varies depending on exploitation of different prey bases and their breeding and migratory phenology, the anthropogenic pressures gannets face in southeastern Newfoundland can differ both intra- and inter-annually. During early chick-rearing (July/early August), gannets typically foraged in coastal waters, while during late chick-rearing adults were less constrained to the coast and extended their foraging distribution towards more pelagic waters. This would place them at greater risk of encountering gillnets associated with inshore cod fishing vessels during early chick-rearing, and more likely to interact with large vessels (e.g., oil tankers) passing through shipping lanes during late chick-rearing (Lieske et al. 2020, Hedd et al. 2015).

Given projections of our warming ocean climate (Oliver et al. 2019), it is likely that marine ecosystems such as the western North Atlantic will experience further shifts in the phenology and distribution of critical prey species (Mbaye et al. 2020, Walsh et al. 2015, Nye et al. 2009), and subsequently, behaviour, foraging distribution and breeding success of marine top predators (Sydeman et al. 2021).

Capelin has already experienced a 30-fold decline in biomass and delayed spawning in the western North Atlantic since the early 1990s. Continual declines in the biomass of capelin may occur as the continuous early sea ice retreat will likely advance the spring bloom and the earlier timing of primary productivity impacting copepods - the key prey of capelin (Buren et al. 2014). Such mismatches may represent a cascade effect along the food web, since the shortage of capelin stocks during the chick-rearing period of the Northern Gannets could hamper the fulfill of energetic requirements of seabirds’ offspring (Buren et al. 2014, 2019). Sea water temperature is the most predictive environmental variable of the horizontal and vertical distribution of capelin from June to November within the study region (e.g., warmer waters linked to decreased presence; Andrews et al. 2020). The importance of sea water temperature on capelin distribution is greatest during August. The influence of warming waters on capelin distribution is critical as it completely overlaps with the chick-rearing period of Northern Gannets breeding at Cape St. Mary’s. If current warming trends remain, capelin distribution could shift northwards and to deeper waters during the breeding season of Northern Gannets, making this prey less available to be captured by parental gannets.

The spawning distribution of Atlantic mackerel is also expected to shift northwards in the coming decades (Mbaye et al. 2020), with a significant overlap likely to occur within the foraging range of Cape St. Mary’s gannets during incubation (May-June) and early (July) chick-rearing period (d’Entremont et al. 2022b). This shift could cause a temporal and spatial mismatch between gannets and mackerel, with mackerel now being available during early chick-rearing, and likely non-existent in late chick-rearing as sea water temperature rise above the species’ critical threshold of ~15-16 °C (Olafsdottir et al. 2019, Montevecchi et al. 2021, d’Entremont et al. 2022a). As mackerel is a critical prey in the diet of Norther Gannets during late chick-rearing (Montevecchi et al. 1984), this shift could have consequences on the reproductive success of gannets whether they are unable to supplement their diet with alternative prey sources in the late chick-rearing. The warming of sea water temperatures in spring/early summer may also trigger a decrease on herring spawning and can induce a shift from spring spawning to mainly autumnal spawning in eastern Newfoundland (Melvin et al. 2009). Such shift would likely change herring abundance throughout the chick-rearing period, from being an important prey during early chick-rearing towards being available during late chick-rearing, which would change the space-use of foraging gannets throughout the different stages of the chick-rearing.

*Conclusions*

Northern Gannets are flexible and opportunistic generalist top predators (Montevecchi et al. 2009). With capelin and herring likely becoming less abundant in early chick-rearing and mackerel likely shifting to be more abundant during early chick-rearing, it is likely that gannets will shift their space-use and prey preferences in Newfoundland as the climate change forces distribution and phenological shifts of their prey. Finally, shifts in prey distributions will likely change the temporal overlap between gannets and fishing vessels and warrants further monitoring in the following years.

**ACKNOWLEDGEMENTS**

We must thank several people for their help with this study. The Cape St. Mary’s Ecological Reserve staff were invaluable, providing accommodations and logistical support over the years. We thank staff member Chris Mooney in particular, for his assistance with tagging gannets in the field. We also thank the Newfoundland and Labrador Parks and Natural Areas Division for allowing us to conduct our research at the Cape St. Mary’s Ecological Reserve. This study was funded by the Natural Sciences and Engineering Research Council and an Ocean and Freshwater Contribution Program grant from Fisheries and Oceans Canada to WAM. Additional funding was provided by NSERC (WAM) and Memorial University of Newfoundland and Labrador. Gannets were handled under the Canadian Wildlife Service permit 10332K and Memorial University of Newfoundland and Labrador animal care permit 19-01-WM.

**AUTHOR CONTRIBUTIONS**

KJND: Conceptualization, data collection, analysis, writing. IP: Conceptualization, analysis, writing. CG: Conceptualization, writing. SNPW: Conceptualization, writing. WAM: Conceptualization, data collection, writing.

**REFERENCES**

Aarts G, MacKenzie M, McConnell B, Fedak M and Matthiopoulos J. Estimating space-use and habitat preference from wildlife telemetry data. Ecography. 2008; 31: 140-160. <https://doi.org/10.1111/j.2007.0906-7590.05236.x>

Andrews S, Leroux SJ, Fortin M-J. Modelling the spatial–temporal distributions and associated determining factors of a keystone pelagic fish. ICES J Mar Sci. 2020; 77 (7-8): 2776-2789.

Brennan CE, Blanchard H, Fennel K. Putting temperature and oxygen thresholds of marine animals in context of environmental change: A regional perspective for the Scotian Shelf and Gulf of St. Lawrence. PLoS ONE. 2016; 11(12): e0167411. doi:10.1371/journal.pone.0167411

Buren AD, Koen-Alonso M, Pepin P, Mowbray F, Nakashima B, Stenson G, Ollerhead N, Montevecchi WA. Bottom-up regulation of capelin, a keystone forage species. PLoS One. 2014; 9:1–11.

Buren, A. D., Murphy, H. M., Adamack, A. T., Davoren, G. K., Koen-Alonso, M., Montevecchi, W. A., et al. The collapse and continued low productivity of a keystone forage fish species. Mar. Ecol. Prog. Ser. 2019; 616: 155–170. doi: 10.3354/meps12924

Burnham KP, Anderson DR. Model selection and multimodel inference, 2nd edn. Springer, New York; 2002.

Calenge C. The package “adehabitat” for the R software: a tool for the analysis of space and habitat use by animals. Ecol Modell. 2006; 197: 516–519.

Carscadden JE, Frank KT, Leggett WC. Ecosystem changes and the effects on capelin (*Mallotus villosus*), a major forage species. Can J Fish Aquat Sci. 2001; 58:73–85.

Carscadden JE, Montevecchi WA, Davoren GK, Nakashima BS. Trophic relationships among capelin (*Mallotus villosus*) and seabirds in a changing ecosystem. ICES J Mar Sci. 2002; 59: 1027-1033.

Carscadden JE, Gjøsæter H, Vilhjálmsson H. A comparison of recent changes in distribution of capelin (Mallotus villosus) in the Barents Sea, around Iceland and in the Northwest Atlantic. Prog Ocean. 2013; 114: 64-83 <https://doi.org/10.1016/j.pocean.2013.05.005>

Crook KA, Maxner E, Davoren GK. Temperature-based spawning habitat selection by capelin (*Mallotus villosus*) in Newfoundland. ICES J Mar Sci. 2017; 74(6): 1622-1629

Davoren GK. Divergent use of spawning habitat by male capelin (*Mallotus villosus*) in a warm and cold year. Beh Ecol. 2013; 24(1): 152-161

d’Entremont KJN, Guzzwell LM, Wilhelm SI, Friesen VL, Davoren GK, Walsh CJ, Montevecchi WA. Northern Gannets (*Morus bassanus*) breeding at their southern limit struggle with prey shortages as a result of warming waters. ICES J Mar Sci. 2022a; 79 (1): 50-60. <https://doi.org/10.1093/icesjms/fsab240>

d’Entremont, K.J.N., Davoren, G.K., Walsh, C.J., Wilhelm, S.I., Montevecchi, W.A. Intra- and inter-annual shifts in foraging tactics by parental Northern Gannets (*Morus bassanus*) in response to changing prey fields. Mar Ecol Prog Ser. 2022b; 698: 155-170. DOI: <https://doi.org/10.3354/meps14164>

DFO. Assessment of the southern Gulf of St. Lawrence (NAFO division 4T-4VN) spring and fall spawner components of Atlantic herring (*Clupea harengus*) with advice for the 2020 and 2021 fisheries. Canadian Science Advisory Secretariat Gulf Region Science Advisory Report 2020/029; 2020.

Dudley SFJ, Field JG, Shelton PA. Distribution and abundance of eggs, larvae and early juveniles of saury *Scomberesox saurus scombroides* (Richardson) off the south-western Cape, South Africa, 1977/78. S Afr J Mar Sci. 1985; 3: 229-237

Elliott KH, Woo K, Gaston AJ, Benvenuti S, Dall’Antonia L, Davoren GK. Seabird foraging behaviour indicates prey type. Mar Ecol Prog Ser. 2008; 354:289–303.

Garthe S, Benvenuti S, Montevecchi WA. Pursuit plunging by Northern Gannets (Sula bassana) feeding on capelin (*Mallotus villosus*). Proc R Soc B. 2000; 267: 1717-1722

Garthe S, Montevecchi WA, Davoren GK. Inter-annual changes in prey fields trigger different foraging tactics in a large marine predator. Limnol Oceanogr. 2011; 56: 802-812

Garthe S, Guse N, Montevecchi WA, Rail J-F, Grégoire F. The daily catch: Flight altitude and diving behavior of northern gannets feeding on Atlantic mackerel. J Sea Res. 2014; 85: 456-462.

Geen GR, Robinson RA, Baillie SR. Effects of tracking devices on individual birds - a review of the evidence. J Avian Biol. 2019; 50: doi:10.1111/jav.01823

Gjerdrum C, Ronconi RA, Turner KL, Hamer, TE. Bird strandings and bright lights at coastal and offshore industrial sites in Atlantic Canada. Avian Conserv Ecol. 2021; 16(1):22. <https://doi.org/10.5751/ACE-01860-160122>

Goyert HF. Relationship among prey availability, habitat, and the foraging behavior, distribution, and abundance of common terns *Sterna* *hirundo* and roseate terns *S.* *dougallii*. Mar Ecol Prog Ser. 2014; 506: 291–302.

Grémillet D, Pichegru L, Kuntz G, Woakes AG, Wilkinson S, Crawford RJM, Ryan PG. A junk-food hypothesis for gannets feeding on fishery waste. Proc R Soc B. 2008; 275: 1149-1156.

Guillemette M, Grégoire F, Bouillet D, Rail J-F, Bolduc F, Caron A, Pelletier D. Breeding failure of seabirds in relation to fish depletion: Is there one universal threshold of food abundance? Mar Ecol Prog Ser. 2018; 587:235-245

Gulka J, Jenkins E, Maynard LD, Montevecchi WA, Regular PM, Davoren GK. Inter-colony foraging dynamics and breeding success relate to prey availability in a pursuit-diving seabird. Mar Ecol Prog Ser. 2020; 651:183-198. <https://doi.org/10.3354/meps13463>

Halpern BS, Walbridge S, Selkoe KA, Kappel CV, Micheli F, D’Agrosa C et al., A global map of human impact on marine ecosystems. Science. 2008; 319, 948–952. 10.1126/science.1149345

Hedd A, Regular PM, Wilhelm SI, Rail J-F, Drolet B, Fowler M et al. Characterization of seabird bycatch in eastern Canadian waters, 1998-2011, assessed from onboard fisheries observer data. Aquat Conserv. 2015; 26(3), 530–548. doi:10.1002/aqc.2551

Hijmans RJ, van Etten J. raster: Geographic analysis and modeling with raster data. R package version 20-12; 2012. <https://cran.r-project.org/web/packages/raster/index.html>

Hipfner J. Matches and mismatches: ocean climate, prey phenology and breeding success in a zooplanktivorous seabird. Mar Ecol Prog Ser. 2008; 368:295–304.

Leim AH, Scott WB. Fishes of the Atlantic coast of Canada. Bull Fish Res Board Can No. 155; 1966.

Lescure L. Foraging flexibility of seabirds (Family Alcidae) under varying prey biomass. Master’s Thesis. University of Manitoba; 2021.

Lieske DJ, McFarlane Tranquilla L, Ronconi RA, Abbott S. Synthesizing expert opinion to assess the at-sea risks to seabirds in the western North Atlantic. Biol Conserv. 2019; 233: 41−50 <https://doi.org/10.1016/j.biocon.2019.02.026>

Lieske DJ, McFarlane Tranquilla L, Ronconi RA, Abbott S. x ‘Seas of risk’: assessing the threats to colonial nesting seabirds in Eastern Canada. Mar Policy. 2019; 115:103863 <https://doi.org/10.1016/j.marpol.2020.103863>

Link JS, Bogstad B, Sparholt H, Lilly GR. Trophic role of Atlantic cod in the ecosystem. Fish Fish. 2009; 10 (1): 58-87.

Luque SP. Diving behaviour analysis in R. R News. 2007; 7: 8-14.

Mbaye B, Doniol-Valcroze T, Brosset P, Castonguay M, Van Beveren E, Smith A, et al. Modelling Atlantic Mackerel spawning habitat suitability and its future distribution in the north-west Atlantic. Fish Oceanogr. 2020; 29:84-99

Melvin GD, Stephenson RL, Power MJ. Oscillating reproductive strategies of herring in the western Atlantic in response to changing environmental conditions. ICES J Mar Sci. 2009; 66 (8): 1784-1792.

Montevecchi WA, Ricklefs RE, Kirkham IR, Gabaldon D. Growth energetics of nestling Northern Gannets (*Sula Bassanus*). Auk. 1984; 101: 334-341.

Montevecchi WA. Binary dietary responses of Northern Gannets (*Sula bassana*) indicate changing food web and oceanographic conditions. Mar Ecol Prog Ser. 2007; 352: 213–220.

Montevecchi WA. Interactions between fisheries and seabirds: Prey modification, discarding and bycatch. In L Young and E Vander Werf (Editors). Conservation of Marine Birds. Academic Press, London U.K; 2023.

Montevecchi WA, Gerrow K, Buren AD, Davoren GK, Lewis KP, Montevecchi MW, Regular PM. Pursuit-diving seabird endures regime shift involving a three-decade decline in forage fish mass and abundance. Mar Ecol Prog Ser. 2019; 627: 171-178.

Montevecchi WA, Regular PM, Rail J-F, Power K, Mooney C, d'Entremont KJN, Garthe S, Guzzwell L, Wilhelm SI. Ocean heat wave induces breeding failure at the southern breeding limit of the Northern Gannet Morus bassanus. Mar Ornithol. 2021; 49: 71-78.

Moores JA, Winters GH, Parsons LS. Migrations and biological characteristics of Atlantic mackerel (*Scomber scombrus*) occurring in Newfoundland waters. J Fish Res Board Can. 1975; 32: 1347-1357

Mowbray TB. Northern Gannet (Morus bassanus), version 1.0. In Birds of the World (S. M. Billerman, Editor). Cornell Lab of Ornithology, Ithaca, NY, USA; 2020. <https://doi.org/10.2173/bow.norgan.01>

Nye JA, Link JS, Hare JA, Overholtz WJ. Changing spatial distribution of fish stocks in relation to climate and population size on the Northeast United States continental shelf. Mar Ecol Prog Ser. 2009; 393, 111–129. <https://doi.org/10.3354/meps08220>

Olafsdottir A. H., Utne K. R., Jacobsen J. A., Jansen T., Óskarsson G. J., Nøttestad L., Elvarsson B. et al. 2019. Geographical expansion of Northeast Atlantic mackerel (Scomber scombrus) in the Nordic Seas from 2007 to 2016 was primarily driven by stock size and constrained by low temperatures. Deep-Sea Res Part II. 2019; 159: 152–168.

Oliver ECJ, Burrows MT, Donat MG, Sen Gupta A, Alexander LV, Perkins-Kirkpatrick SE et al. Projected marine heatwaves in the 21st century and the potential for ecological impact. Front Mar Sci. 2019; https://doi.org/10.3389/fmars.2019.00734

Penton PM, Davoren GK. Physical characteristics of persistent deep-water spawning sites of capelin: Importance for delimiting critical marine habitats. Mar Biol Res. 2012; 8: 778-783.

Pelletier D, Guillemette M. Times and partners are a-changin’: Relationships between declining food abundance, breeding success, and divorce in a monogamous seabird species. Peer J. 2022; 10:e13073 <https://doi.org/10.7717/peerj.13073>

Piatt JF. The aggregative response of common murres and Atlantic puffins to their prey. Stud Avian Biol. 1990; 14:36–51.

Plourde S, Grégoire F, Lehoux C, Galbraith PS, Castonguay M, Ringuette M. Effect of environmental variability on body condition and recruitment success of Atlantic Mackerel (Scomber 2014; scombrus L.) in the Gulf of St. Lawrence. Fish Oceanogr. 2015; 24: 347-363

Raine AF, Gjerdrum C, Pratte I, Madeiros J, Felis JJ, Adams J. Marine distribution and foraging habitat highlight potential threats at sea for the Endangered Bermuda petrel Pterodroma cahow. Endang Species Res. 2021; 45:337-356. <https://doi.org/10.3354/esr01139>

Regular PM, Hedd A, Montevecchi WA, Robertson GJ, Storey AE, Walsh CJ. Why timing is everything: Energetic costs and reproductive consequences of resource mismatch for a chick-rearing seabird. Ecosphere. 2014; 5 (12): 1-13.

Robertson GJ, Wiese FK, Ryan PC, Wilhelm SI. Updated numbers of murres and dovekies oiled in Newfoundland waters by chronic ship-source oil pollution. In Proceedings of the 37th AMOP Technical Seminar on Environmental Contamination and Response (pp. 265-275). Environment Canada Ottawa, ON; 2014

Smith A, Van Beveren E, Girard L, Boudreau M, Brosset P, Castonguay M, Plourde S. Atlantic mackerel (*Scomber scombrus* L.) in NAFO Subareas 3 and 4 in 2018. Canadian Science Advisory Secretariat (CSAS) Research Document 2020/013 Quebec Region; 2020.

Sydeman WJ, Schoeman DS, Thompson SA, Hoover BA, Garcia-Reyes M, Daunt F, et al. Hemispheric asymmetry in ocean change and the productivity of ecosystem sentinels. Science. 2021; 372 (6545): 980-983. DOI: 10.1126/science.abf1772

Walsh HJ, Richardson DE, Marancik KE, Hare JA. Long-term changes in the distributions of larval and adult fish in the northeast US shelf ecosystem. PLoS one. 2015; 10(9), e0137382. <https://doi.org/10.1371/journal.pone.0137382>

Wilhelm SI, Mailhiot J, Arany J, Chardine JW, Robertson GJ, Ryan PC. Update and trends of three important seabird populations in the western North Atlantic using a geographic information system approach. Mar Ornithol. 2015; 43: 211-222.

Wilhelm SI, Dooley SM, Corbett EP, Fitzsimmons MG, Ryan PC, Robertson GJ. Effects of land-based light pollution on two species of burrow-nesting seabirds in Newfoundland and Labrador, Canada. Avian Conserv Ecol. 2021; 16(1):12. <https://doi.org/10.5751/ACE-01809-160112>

Wood SN. mgcv: Mixed GAM computation vehicle with automatic smoothness estimation. R package version 1.8-38; 2021. <https://cran.r-project.org/web/packages/mgcv/mgcv.pdf>
