# Supplementary material for: Quantifying inter-annual variability on the space-use of parental Northern Gannets (Morus bassanus) in pursuit of different prey types
Source: PLoS One. 2023 Jul 14;18(7):e0288650. doi: 10.1371/journal.pone.0288650 (PMC10348513; doi:10.1371/journal.pone.0288650)
Supplement: Attachment — Submitted filename: Response to Reviewers_PlosOne Resubmission.docx [file pone.0288650.s002.docx]

**PLEASE NOTE: All responses to reviewer questions have been added in GREEN.**

PONE-D-23-00624

Quantifying space-use of parental Northern Gannets (Morus bassanus) in pursuit of different prey types

PLOS ONE

Dear Dr. d'Entremont,

Thank you for submitting your manuscript to PLOS ONE. After careful consideration, we feel that it has merit but does not fully meet PLOS ONE’s publication criteria as it currently stands. Therefore, we invite you to submit a revised version of the manuscript that addresses the points raised during the review process.

Please submit your revised manuscript by Apr 14 2023 11:59PM. If you will need more time than this to complete your revisions, please reply to this message or contact the journal office at [plosone@plos.org](mailto:plosone@plos.org). When you're ready to submit your revision, log on to <https://www.editorialmanager.com/pone/> and select the 'Submissions Needing Revision' folder to locate your manuscript file.

Please include the following items when submitting your revised manuscript:

- A rebuttal letter that responds to each point raised by the academic editor and reviewer(s). You should upload this letter as a separate file labeled 'Response to Reviewers'.
- A marked-up copy of your manuscript that highlights changes made to the original version. You should upload this as a separate file labeled 'Revised Manuscript with Track Changes'.
- An unmarked version of your revised paper without tracked changes. You should upload this as a separate file labeled 'Manuscript'.

If you would like to make changes to your financial disclosure, please include your updated statement in your cover letter. Guidelines for resubmitting your figure files are available below the reviewer comments at the end of this letter.

If applicable, we recommend that you deposit your laboratory protocols in protocols.io to enhance the reproducibility of your results. Protocols.io assigns your protocol its own identifier (DOI) so that it can be cited independently in the future. For instructions see: [https://journals.plos.org/plosone/s/submission-guidelines#loc-laboratory-protocols](https://can01.safelinks.protection.outlook.com/?url=https%3A%2F%2Fjournals.plos.org%2Fplosone%2Fs%2Fsubmission-guidelines%23loc-laboratory-protocols&data=05%7C01%7CCarina.Gjerdrum%40ec.gc.ca%7C04cf68f8a99546495b3f08db19b8e968%7C740c5fd36e8b41769cc9454dbe4e62c4%7C0%7C0%7C638132054679704643%7CUnknown%7CTWFpbGZsb3d8eyJWIjoiMC4wLjAwMDAiLCJQIjoiV2luMzIiLCJBTiI6Ik1haWwiLCJXVCI6Mn0%3D%7C2000%7C%7C%7C&sdata=ZFq20kORldh3FVz%2BVZzJGBYcBHtfTRay6kn2oCVB55A%3D&reserved=0). Additionally, PLOS ONE offers an option for publishing peer-reviewed Lab Protocol articles, which describe protocols hosted on protocols.io. Read more information on sharing protocols at [https://plos.org/protocols?utm_medium=editorial-email&utm_source=authorletters&utm_campaign=protocols](https://can01.safelinks.protection.outlook.com/?url=https%3A%2F%2Fplos.org%2Fprotocols%3Futm_medium%3Deditorial-email%26utm_source%3Dauthorletters%26utm_campaign%3Dprotocols&data=05%7C01%7CCarina.Gjerdrum%40ec.gc.ca%7C04cf68f8a99546495b3f08db19b8e968%7C740c5fd36e8b41769cc9454dbe4e62c4%7C0%7C0%7C638132054679704643%7CUnknown%7CTWFpbGZsb3d8eyJWIjoiMC4wLjAwMDAiLCJQIjoiV2luMzIiLCJBTiI6Ik1haWwiLCJXVCI6Mn0%3D%7C2000%7C%7C%7C&sdata=yVn1u00vp%2B2f7gaTcI1JxGqNv7yinzdb%2FADZ%2FHopGgo%3D&reserved=0).

We look forward to receiving your revised manuscript.

Kind regards,

Vitor Hugo Rodrigues Paiva, Ph.D.
Academic Editor
PLOS ONE

Journal Requirements:

When submitting your revision, we need you to address these additional requirements.

1. Please ensure that your manuscript meets PLOS ONE's style requirements, including those for file naming. The PLOS ONE style templates can be found at 
<https://journals.plos.org/plosone/s/file?id=wjVg/PLOSOne_formatting_sample_main_body.pdf> and 
<https://journals.plos.org/plosone/s/file?id=ba62/PLOSOne_formatting_sample_title_authors_affiliations.pdf>

2. Thank you for stating the following in the Acknowledgments Section of your manuscript: 
   "We must thank several people for their help with this study. The Cape St. Mary’s Ecological Reserve staff were invaluable, providing accommodations and logistical support over the years. We thank staff member Chris Mooney in particular, for his assistance with tagging gannets in the field. We also thank the Newfoundland and Labrador Parks and Natural Areas Division for allowing us to conduct our research at the Cape St. Mary’s Ecological Reserve. This study was 
funded by the Natural Sciences and Engineering Research Council and an Ocean and Freshwater Contribution Program grant from Fisheries and Oceans Canada to WAM. Additional funding was provided by NSERC (WAM) and Memorial University of Newfoundland and Labrador. Gannets were handled under the Canadian Wildlife Service permit 10332K and Memorial University of Newfoundland and Labrador animal care permit 19-01-WM."

We note that you have provided funding information that is not currently declared in your Funding Statement. However, funding information should not appear in the Acknowledgments section or other areas of your manuscript. We will only publish funding information present in the Funding Statement section of the online submission form.

Please remove any funding-related text from the manuscript and let us know how you would like to update your Funding Statement. Currently, your Funding Statement reads as follows:

  "WAM received grant #2018-06872 from the Natural Sciences and Engineering Research Council of Canada Discovery Grant program. The funder played no role in study design or the publication process. URL: <https://www.nserc-crsng.gc.ca/professors-professeurs/grants-subs/dgigp-psigp_eng.asp>

WAM received the sub-grant # 57177 to Memorial University of Newfoundland and Labrador from the Fisheries and Oceans Canada Coastal Environmental Baseline Program. The funder played no role in study design or the publication process. URL: <https://www.dfo-mpo.gc.ca/science/partnerships-partenariats/research-recherche/cebp-pdecr/index-eng.html>"

Please include your amended statements within your cover letter; we will change the online submission form on your behalf.

All funding information has been removed from the Acknowledgements section in the manuscript.

3. In your Data Availability statement, you have not specified where the minimal data set underlying the results described in your manuscript can be found. PLOS defines a study's minimal data set as the underlying data used to reach the conclusions drawn in the manuscript and any additional data required to replicate the reported study findings in their entirety. All PLOS journals require that the minimal data set be made fully available. For more information about our data policy, please see <http://journals.plos.org/plosone/s/data-availability>.

"Upon re-submitting your revised manuscript, please upload your study’s minimal underlying data set as either Supporting Information files or to a stable, public repository and include the relevant URLs, DOIs, or accession numbers within your revised cover letter. For a list of acceptable repositories, please see <http://journals.plos.org/plosone/s/data-availability#loc-recommended-repositories>. Any potentially identifying patient information must be fully anonymized.

Important: If there are ethical or legal restrictions to sharing your data publicly, please explain these restrictions in detail. Please see our guidelines for more information on what we consider unacceptable restrictions to publicly sharing data: <http://journals.plos.org/plosone/s/data-availability#loc-unacceptable-data-access-restrictions>. Note that it is not acceptable for the authors to be the sole named individuals responsible for ensuring data access.

We will update your Data Availability statement to reflect the information you provide in your cover letter.

The entire minimal underlying dataset pertinent for this manuscript is now publicly available in a data package on the biotelemetry data repository “MoveBank” at the following DOI: <https://doi.org/10.5441/001/1.5km7v2s3>

4. Please include your full ethics statement in the ‘Methods’ section of your manuscript file. In your statement, please include the full name of the IRB or ethics committee who approved or waived your study, as well as whether or not you obtained informed written or verbal consent. If consent was waived for your study, please include this information in your statement as well.

Added the following to L463: “Gannets were handled under the Canadian Wildlife Service permit 10332K and Memorial University of Newfoundland Animal Care Committee animal care permit 19-01-WM. “

5. We note that Figure 1 in your submission contain [map/satellite] images which may be copyrighted. All PLOS content is published under the Creative Commons Attribution License (CC BY 4.0), which means that the manuscript, images, and Supporting Information files will be freely available online, and any third party is permitted to access, download, copy, distribute, and use these materials in any way, even commercially, with proper attribution. For these reasons, we cannot publish previously copyrighted maps or satellite images created using proprietary data, such as Google software (Google Maps, Street View, and Earth). For more information, see our copyright guidelines: <http://journals.plos.org/plosone/s/licenses-and-copyright>.
 
We require you to either (a) present written permission from the copyright holder to publish these figures specifically under the CC BY 4.0 license, or (b) remove the figures from your submission:
 
a. You may seek permission from the original copyright holder of Figure 1 to publish the content specifically under the CC BY 4.0 license.  
 
We recommend that you contact the original copyright holder with the Content Permission Form (<http://journals.plos.org/plosone/s/file?id=7c09/content-permission-form.pdf>) and the following text:
“I request permission for the open-access journal PLOS ONE to publish XXX under the Creative Commons Attribution License (CCAL) CC BY 4.0 (<http://creativecommons.org/licenses/by/4.0/>). Please be aware that this license allows unrestricted use and distribution, even commercially, by third parties. Please reply and provide explicit written permission to publish XXX under a CC BY license and complete the attached form.”
 
Please upload the completed Content Permission Form or other proof of granted permissions as an "Other" file with your submission.
 
In the figure caption of the copyrighted figure, please include the following text: “Reprinted from [ref] under a CC BY license, with permission from [name of publisher], original copyright [original copyright year].”
 
b. If you are unable to obtain permission from the original copyright holder to publish these figures under the CC BY 4.0 license or if the copyright holder’s requirements are incompatible with the CC BY 4.0 license, please either i) remove the figure or ii) supply a replacement figure that complies with the CC BY 4.0 license. Please check copyright information on all replacement figures and update the figure caption with source information. If applicable, please specify in the figure caption text when a figure is similar but not identical to the original image and is therefore for illustrative purposes only.
The following resources for replacing copyrighted map figures may be helpful:
 
USGS National Map Viewer (public domain): <http://viewer.nationalmap.gov/viewer/>
The Gateway to Astronaut Photography of Earth (public domain): <http://eol.jsc.nasa.gov/sseop/clickmap/>
Maps at the CIA (public domain): <https://www.cia.gov/library/publications/the-world-factbook/index.html> and <https://www.cia.gov/library/publications/cia-maps-publications/index.html>
NASA Earth Observatory (public domain): <http://earthobservatory.nasa.gov/>
Landsat: <http://landsat.visibleearth.nasa.gov/>
USGS EROS (Earth Resources Observatory and Science (EROS) Center) (public domain): [http://eros.usgs.gov/#](http://eros.usgs.gov/)
Natural Earth (public domain): <http://www.naturalearthdata.com/>

[Note: HTML markup is below. Please do not edit.]

Figure 1 was constructed with publicly available data, namely bathymetry from GEBCO from which we extracted isobath, and GADM shapefile of North America, which is also a publicly available layer. Can you provide further details as to why you believe it contains copyrighted material? We don’t see images in it that might be copyrighted.

**5. Review Comments to the Author**

**Reviewer #1**:

Authors,

I enjoyed reading this study about how habitat use models differ based on which behaviour (in this case dive type) is used as a response variable. I think the premise is highly worthwhile, and using the behaviour of gannets to infer the availability of different forage fishes based on foraging mode is really interesting. However, I have some concerns about the methodology and surrounding narrative that I believe need to be addressed. See below for specific comments.

**L44-46**: This sentence is too complex, maybe split into two, with the latter explaining the depleted forage fish stocks.

Changed “overexploitation by the fisheries industry” to “overfishing” to improve readability.

**L62:** Maybe “slowing” instead of “asymptotic” might read a little easier.

Changed to: ” and plateaued population size”.

**L101-104:** This section should be represented as a table of deployments.

Thank you for this suggestion, but we kept the sentence as is and believe the sentences we have provided in our manuscript succinctly summarizes our device deployments.

**L120-122:** This is a bit confusing: What happens if a bird dives for > 10s and has a bottom time of < 3s? Could just go by Cox et al. (2016) and solely use bottom time thresholds to separate dive types?

In the instance you are describing, the dive would be designated as a “U-dive”, as it meets one of the criteria, being greater than 10 s. We used the criteria as described in the text to remain consistent with Garthe et al. 2000 and d’Entremont et al. 2022.

**L142-143**: I agree that the two types of dive profiles may reflect availability of different prey types, however, this then raises the question of why V-shaped dives were split into early and late?

To provide greater clarity for this split, we have added the following to L130: “Although U-shaped dives peak during early chick-rearing, V-shaped dives are performed by gannets throughout the chick-rearing period. To account for the differing prey species in the region in early and late chick-rearing and the possibility that early V-shaped dives could be associated with herring rather than mackerel, or singular capture of capelin (rather than a U-shaped dive for multiple fish), the potential influence U-shaped dive behaviour might have on the occurrence and distribution of V-shaped dives, we divided V-shaped dives in two categories: early and late V-shaped dive.”

U-shaped dives also occur at almost all times?

U-shaped dives by parental gannets ranging from Cape St. Mary’s are significantly less likely to occur past mid-August. Please see Figure 2 from d’Entremont et al. 2022b: <https://doi.org/10.3354/meps14164>. Also, it is key to note that U-dives are explicitly associated with capelin owing to swimming speeds of key prey species (e.g., U-dives are too slow for gannets to capture species other than capelin in this ecosystem) whereas V-dives can be associated with multiple species

You mention earlier that U-shaped dives may influence V-shaped dives, but I’m not sure this is really logical. You might need to stick to two different responses (which I would personally opt for), early and late for both dive types, or find stronger justification for the three current responses.

Please see above commentary regarding the decision to split V dives into early and late.

**L152-155:** I don’t agree with the use of pseudo-absences, when perfectly good absences exist. Why not use locations with no associated dives as the absence in your response? Because dive behaviour is your response, the presence or absence of that behaviour along the track should form your response variable, rather than randomly generated pseudo-absences.

We understand the comment. We used pseudo-absences (“pseudo” because we don't truly have absence information for every location within our study area) mostly because we were interested in foraging distribution and not the driver of individual behaviour. Although, that would be a very interesting question. Considering the "real" absence along the track would be an interesting approach if we wanted to look at drivers of individual behaviour, but our question was rather to consider the diving behaviour of gannet as a proxy for prey distribution.

**L157:** What’s the temporal resolution of SST? Is it MODIS data, and if so, is it level 3 or level 4 type data?

It is Level 4 MODIS data at 0.01° spatial resolution, daily. See newly added sentence regarding the source of our SST data on L177: “SST data were retrieved from the Multi-scale Ultra-high Resolution SST Analysis fv04.1 dataset on the Environmental Research Division’s Data Access Program (ERDDAP, https://coastwatch.pfeg.noaa.gov/erddap/griddap/jplMURSST41.html).”

**L158:** Why not latitude? It may be colinear with SST, but probably worth considering in any case. It would be good to include a 2D lat/lon spline.

We added latitude to our model in a 2D lat/lon spline as suggested by the reviewer.

**L167:** Whole model selection using MuMIn::dredge or similar would be much better if feasible.

Thanks to the reviewer's advice, we did not know about the MuMIn::dredge function. We followed their advice and used this function for our model selection, even though it barely o the final model selected with 10-fold cross-validation used before.

**L170-171:** The mgcv::concurvity function is a neat way of looking for multicollinearity within your model. This way is okay too, but the other would be better.

Thank you for informing us of this function! We are now checking for concurvity, which led us to remove dist2col from our models especially now that we are using 2D spline with Lat,Lon.

**L180-181:** If V-shaped dives are being split into early and late, surely they should only be predicted over the time span they are associated with? Please clarify if this is the case or rectify if not.

Added the following for clarity: “(predicted within the respective time spans for early and late V-dives)” to L208.

**L188:** Maybe association is incorrect here? “Temporal variation in dive types” instead?

Changed to: “There was a significant temporal variation in the distributions of U- and V-shaped dives among years (*χ^2^* = 122.78, *p* < 0.001).”.

**L202-204:** This spatial variation between early and late V-shaped dives could potentially be good justification for splitting into two responses. However, not sure the pattern is strong enough.

Please see above commentary regarding the decision to split V dives into early and late. This can be found in response to your original comment regarding L142-143.

**Table 2:** Models with nothing but “distance to colony” returning an AUC of > 0.9 is a case in point for why pseudo-absences are a poor substitute for genuine absences when trying to determine environmental drivers of behaviour, and not solely distribution. This is evident when adding other environmental variables has a negligible impact on model AUC.

Thanks for this feedback, this is an interesting question to look at drivers of behaviour. However, as mentioned previously, we did not for this paper, focus on gannet individual behaviours and what can be a driver of it, but rather we looked at how to use the habitat they do select as a potential tool to predict prey distribution.

Also, now that we are using 2D splines with Latitude and Longitude, we decided to remove distance to the colony from our models. Notably because after checking concurvity in our model smooth terms, it was clear that dist2col and 2D Lat,Lon could not be used in the same model, they were highly collinear.

As per the suggestion above, we decided to use the MuMln::*dredge* function to decide which model would be our model for habitat suitability prediction. This function iteratively evaluates all possible models from a global model and its suite of covariates; the final model is selected based on AICc; we thus changed our method and are not using AUC anymore.

**L242-257 & figure 5**: Model covariates appear a little bit overfit in some cases. In general, you should discuss the model specification in a bit more detail. Are you using cubic or thin-plate splines? Is any shrinkage applied? Shrinkage would be a tidy way to reduce instances of overfitting and return the simplest effective spline. Perhaps transforming heavily skewed variables, such as slope and bathymetry, could be beneficial too, as this might reduce the huge uncertainty around extreme values.

For bathymetry, and slope, we corrected the shape of the polygon used to extract the values to make certain only values < 0 m, over the ocean, were included. We now specified that we used penalized thin-plate regression splines, and let the model select the number of knots to attribute to the smoother. We believe our models are now improved, thanks to the reviewers suggestions.

**Figure 5**: All the bathymetry plots have positive values (assumably on land) with a very high likelihood of dives occurring. Might need to look at how the data are prepared, figure out why this happening, and fix it.

We fixed the Bathymetry layer. The NOGA Domain polygon was not based on the GEBCO bathymetry layer, thus the land boundaries were not exactly aligning together. We fixed this by using the GEBCO bathymetry layer to define the land boundaries of the NOGA Domain polygon in which are generated the random locations at which depth values are extracted. Thanks for pointing this out to us.

**Figure 5**: I would also highly recommend the mgcViz package for plotting model outputs from mgcv.

This is the package that we used to plot the model outputs. However, we went further by customizing the visual of the figure; more functionalities can be achieved with mgcViz, and maybe this is why it does not look (visually) like the default mgcViz plot.

**L275-283**: Really interesting links here between dive type and prior expectation of the availability of prey. Seems that if a prey type is lacking, they increase the associate dive effort rather than concentrating on other dive types/prey species. Could potentially expand into temporal changes in dietary requirements and why this might prevent plasticity, or might be too complex a topic to just touch on. Just a thought.

Added the following to the end of this paragraph: “As capelin is a key prey species for gannets during early chick-rearing due to the size and energetic requirements of the chick (Montevecchi et al. 1984), this might explain why U-dives were greater than expected when capelin availability was low in 2020. This reliance on capelin in early chick-rearing due to energetic and size (e.g., prey larger than the smaller capelin may not be consumable by the smaller chicks at this stage) constraints of the chick might prevent foraging plasticity at this critical time period. “

**L306-308, L321, L370-371**: These sentences contradict each other about the temperature preferences of mackerel, or at least seem to?

The temperature threshold of greater than 18°C that was originally on L321 was in reference to saury only. As such, it has now been deleted.

**L346-350**: It could be good here to foreshadow the extra energy that gannets may have to expend to target desired prey species? You have a basis for this, given the additional effort in U-shaped dives in the year when capelin was less abundant, and increases in V-shaped dive effort in years with fewer saury and mackerel around. Otherwise, this paragraph seems a bit light and vague for quite an important and relevant point.

Added the following to L395: “ With these shifts in distribution and phenology, it is expected that gannets will have to expend greater energy for foraging to compensate for these changes in availability, as has already been seen in the Northwest Atlantic during years of poor capelin availability (d’Entremont et al. 2022b).”

Also tempered this statement further down by adding the following to L412: “However, as gannets exhibit foraging plasticity within and between years (e.g. Pettex et al. 2012, d’Entremont et al. 2022b), they may be able to compensate for these distributional shifts by exploiting other prey species. Further monitoring of the foraging behaviour of parental gannets at Cape St. Mary’s is warranted to assess responses to these distributional shifts. “

**L381-387**: The conclusion as a whole does not reflect the research that has gone into this paper. Please rewrite, with a focus on the actual findings instead of projecting how space-use will change, which you haven’t really shown.

The conclusion has been re-written to more accurately reflect our study.

I hope you find these comments useful.
All the best

**Reviewer #2:**

First of all, I want to congratulate the authors for the great work performed to study the habitat use and spatial distribution of the northern gannet (Morus bassanus) by taking into account their two major diving tactics. Overall, the authors put a considerable amount of effort for habitat suitability modeling exercises, quite complex analyses, and I commend them for this.

Nevertheless, I have a major concern about the modeling approach and some other minor/medium questions that should be taken into consideration before the final approval.

I have added my comments in the word version using track changes, pointing some typographical or grammatical errors, but I provide here the main issues:

**INTRODUCTION:**

**L117:** I feel that in this paragraph (L117-L128) should be included more information about the use of habitat suitability models in seabirds. Perhaps authors could indicate some studies where they were successfully applied, even in gannets or other Sulidae species.

Added the following: “Habitat suitability models have been used broadly to identify critical habitat for several seabird species (e.g., Raine et al. 2021, Häkkinen et al. 2021, Skov et al. 2008). “

**L130:** Perhaps the ideas in this sentence are in the reverse order. The dive profiles could change when the foraging areas are different, due to the foraging habitat conditions (depth, slope, etc) and to the prey inhabiting these different habitats.

Thanks for this suggestion, however we have left the ordering as is. The main objective we had was to assess if space-use differed when using different dive profiles.

**METHODS:**
**L215:** I am concerned about the computation for the estimation of the smoothing parameter (h). Href is a low-conservative method, so possibly we can be dealing with a considerable error that will affect kernel density estimation. I recommend the use of more recent methodologies, such as those in Lascelles et al., 2016 that provide a h value more adapted to your own data.

Lascelles BG, Taylor PR, Miller MGR, Dias MP, Oppel S, Torres L, Hedd A, Le Corre M, Phillips RA, Shaffer SA, Weimerskirch H, Small C (2016) Applying global criteria to tracking data to define important areas for marine conservation. Divers Distrib 22:422–431. doi: 10.1111/ddi.12411

We understand the method developed by Lascelles et al. 2016 for having used it elsewhere. Lascelles et al. (and the method now applicable through the track2KBA package - Beal et al. 2021) consider an individual trip at sea as the unit of measurement to estimate the smoothing factor *h*. Thus the scale determined with their method does not necessarily correspond to the scale of the dive locations distribution which are extracted from the individual trip; such as 95% UD with the small *h* = 4 km (log of the average foraging range) produced using the function *findScale* from the track2KBA package (Beal et al. 2021) produced polygon too constricted around the dive locations instead of being more unified in whole as should be the 95% distribution i.e., the range of the distribution.

However, we cannot use the Lascelles et al. method directly on diving locations considered on their own as the method considers individual trips to determine the scale of movement to compute *h* parameter that pertains to the movement scale of animals.

Nonetheless, and in response to the comment related to the potentially “low-conservative” output of using *href*, we lowered the smooth *h* that we used by increasing the *h* value starting from the value *mag* namely the log of the average foraging range (out from the function *findScale* in track2KBA) and multiplied it by 2 to achieve better coverage considering the 95% UD and to adjust the smoothing parameter according to reasonable space use of the animals (Beal et al. 2021).

The purpose of using the kernel estimator in our case is more as a way to visually represent the distribution of the different dive shapes. In regard to those considerations, we believe our representation is improved by being less conservative and in tune to the dive patterns of the animals.

**L241-267:** Once the individuals performed several dives, I recommend the use of individual as a random factor to avoid pseudo-replication issues. I mean, perhaps an additive mixed model might be more adequate to analyze the data.

Indeed, individuals may have different habitat preferences, and fitting random slopes for individuals might be useful, however mixed effects models do not predict into new datasets when random effect levels differ. Thus, adding individuals as random effects is difficult given our goal of developing predictive models based on presence and pseudo-absence locations, which are not associated with individuals. We added those precision to our MS in the methods.

Additionally, there are some other gaps that should be clarified in this section:

- Please refer how the pseudo-absences were generated prior modeling exercises.

Added the following on L172: “using the “spsample” function in the R package “sp” (Pebesma and Bivand 2005,”

- It seems that the “year” was not included in the modeling exercises. Since part of this manuscript was focused on the inter-annual variability on the space-use, the “year” must be included as a covariate.

We added Year as a fixed term in the modeling exercise.

- Have you used smoothed terms in GAM? Perhaps by smoothing SST may be a good way to deal with possible non-linear. distribution of the variable.

See comment directly below.

- Finally, if smooth terms were used please refer what was the number of thin plate regression splines.

Yes we did use each covariate as a smooth term. Now this section reads like: “For each dive type, we determined which variables to include in our final model by constructing a generalized additive model (GAM) with each variable included as the smooth parameters of the model and using penalized thin-plate regression spline. Latitude and longitude were smoothed using a two dimensional splines.”

- Besides correlation among the covariates, test for multicollinearity should be evaluated (for instance using the variation inflation factor, VIF), and concurvity if smoothed terms were used.

Two good guides for analyzing ecological data:
Zuur AF, Ieno EN, Smith GM (2007) Analysing ecological data. Springer, New York

Morlini I (2006) On multicollinearity and concurvity in some nonlinear multivariate models. Stat Methods Appl 15:3–26. <https://doi.org/10>. 1007/s10260-006-0005-9

Thank you so much for this comment. Now that we are using 2D spline with Latitude and Longitude, we removed Distance to the Colony from our models. Notably because after checking concurvity in our model smooth terms, it was clear that dist2col and 2D Lat,Lon could not be used in the same model, they were highly collinear.

**-** Have you used AIC or the ΔAICc? The more correct is using the ΔAICc (the difference in AICc between a given model and the model with the smallest AICc). Nevertheless, I suggest the use of AIC corrected for small sample sizes (AICc), because you were working with a low number of individuals.

Burnham KP, Anderson DR (2002) Model selection and multimodel inference: a practical information-theoretic approach. Springer- Verlag, New York

Now that we have changed the way the final model selection was achieved, we added a table with the top candidate models from the MuMl::dredge function, and displayed their AICc, delta AIC, and weight.

Additive models are quite useful when working with non-linear variables, such as environmental predictors, however, some assumptions must be fulfilled before model runs.

GAM models assume independence of error, which is difficult to achieve considering autocorrelation is inherent to tracking data. However, given our predictive goal associated with building those GAMs, we could not consider adding random effects, which does not enable prediction into a new dataset.

We performed a model check and our models were acceptable according to the residual distribution, and the linearity of the quantiles of the deviance of the residuals (qqplot), but assume that these are only indicative as GAM can be more complicated.

A good book/guide for the mixed models:

Zuur AF, Ieno EN,WalkerN, Saveliev A a., SmithGM (2009) Mixed effects models and extensions in ecology with R. Springer, New York


**RESULTS:**
**L367:** In fact, the threshold is not that clear for me, according to Figure 5a. I would rather say that the U-shaped dives probability of occurrence decreased beyond 150 km from the colony, rather than 200 km.

We adjusted our result section according to our updated models and figures. Distance from the colony was removed from our updated models due to concurvity with other smoothing terms.

**L368:** On the other hand, the early V-shaped dives seemed to decrease beyond 200 km rather than 150 km, while the late V-shaped dives have a strong negative relationship with distance to the colony; at least until 200 km.

See above comment.

**L370-371:** This was more evident for U-shaped dives than for late V-shaped dives. The effect that authors highlight here is not that clear to me. I believe that may be some real effect on U-shaped dives, however, for V-shaped dives the effect is visually weak.

We adjusted our result section according to our updated models and figures. The results read a bit differently now, and we note when the effects are weak or slim. Indeed, the overall model was best fitted for U-shaped than for both early and late V-shaped dive, likely because more than one species of prey are targeted during V-shaped dive which might lead gannets to use a larger type of habitats than when foraging on capelin using U-shaped dives.

**DISCUSSION:**

After reading the discussion it feels like some studies may have been left behind. I recommend the inclusion of concrete examples in northern gannets or in other gannet species or even boobies. Despite being different species, they have similar foraging and feeding behavior tactics when foraging, thus it can improve substantially the discussion of your results. Moreover, according to the results, it seems quite important the habitat characteristics (e.g., slope) on determining the diving behavior, however, some studies reported that oceanographic conditions can be preponderant as well.

Some possible suggestions:

Cox SL, Miller PI, Embling CB, Scales KL, Bicknell AWJ, Hosegood PJ, Morgan G, Ingram SN, Votier SC. 2016 Seabird diving behaviour reveals the functional significance of shelf-sea fronts as foraging hotspots. R. Soc. open sci. 3: 160317. <http://dx.doi.org/10.1098/rsos.160317>

Cleasby IR, Wakefield ED, Bodey TW, Davies RD, Patrick SC, Newton J, Votier SC, Bearhop S, Hamer KC (2015) Sexual segregation in a wide-ranging marine predator is a consequence of habitat selection. Mar Ecol Prog Ser 518:1–12. https:// doi. org/ 10. 3354/ meps1 1112

Additional references regarding Cape and Australasian Gannets have been added to the Discussion.

Angel LP, Barker S, Berlincourt M, Tew E, Warwick-Evans V, Arnould JPY. Eating locally: Australasian gannets increase their foraging effort in a restricted range. Biology open. 20

Thiebault A, Semeria M, Lett C, Tremblay Y. How to capture fish in a school? Effect of successive predator attacks on seabird feeding success. J Anim Ecol. 2016; 85, 157-167. https://doi.org/10.1111/1365-2656.1245515;4(10):1298–305. pmid:26369928

**L399:** "We found the number of U-shaped dives was greater than expected in 2020." Please avoid this type of sentences in the discussion. This is already stated in the results.

Statement removed.

**L639-640:** Another important variable that might interest the authors might be the distance travelled between two dives. I am just wondering that if adults dive more frequently and used the same diving tactic can be a good indicator of the distribution of prey among and within years. I do not know if your small database (10, 7, and 8 individuals each year) would allow to test this, however it can be very informative. It may help to explain the inter-annual variability in the spatial use of habitat by gannets.

However, this is not something we could test here because the data distribution includes the randomized pseudo-absence which does not enable us to generate such data. Again, a great point to look at drivers of individual behaviour.

Please check the track changes in the word file. I added some minor comments there and other suggestions that I hope have improved the quality of the manuscript.

**Reviewer #3**:

Thanks to the authors for an interesting and well-written study. The study investigates suitable habitats of forage fishes inferred from the different dive profiles performed by chick-rearing gannets. It identifies important gannet foraging areas and several of their key environmental features using animal-borne biologging data collected over multiple years.

The authors propose that a subset of these areas be considered as critical areas of multi-species conservation concern that should be prioritized in protection efforts. In general, the objectives of the study as set out by the authors are achieved and I have no major concerns. Suggestions for improvement are given below.

Please provide higher resolution versions of all figures, especially 3 and 4, these are largely illegible due to blurriness. Consider in figures 3 and 4 to differentiate between land and isobath lines more clearly, for example by using darker grey for land. In addition in figure 3, some panels seem to cut off the southern edge of the distributions.

High resolution figures were included in the submission, as separate files. It appears the quality was only degraded when automatically included in the generated pdf created upon submission.

**Line 20:** No need to capitalize “Kernel Density”, change to “kernel density”.

Done.

**Line 33:** Replace “incidence on” with “consequences for”.

Done.

**Line 46**: Consider listing one reference per effect (i.e. one for overexploitation by fisheries, one each for climate-induced shifts) to avoid over-referencing.

We feel that the appropriate references have been cited for this paragraph.

**Line 82**: Unclear why “cold-blooded” is relevant here, suggest either deleting or inserting clarification earlier (e.g. at line 77).

Deleted “cold-blooded”.

**Line 90**: d’Entremont et al. 2022 a, b or both?

Fixed to “2022a”.

**Line 114-115**: Assuming devices set to record GPS locations and dive depth. Also please clarify what the range of the base station was.

Changed phrasing to : “Devices were set to only record GPS locations and dive information when out of range of the base station to conserve battery life.”

Added the following to L116: “ The detection range of the base station varies from 200-500 m, depending on environmental conditions.”

**Line 117**: Somewhere at beginning of data processing/analysis please state what software was used (R and R Studio?), and which version.

Added statement addressing this on L122.

**Line 123-125**: Please include a justification (i.e. a reference) for foraging location annotation based on within 30 minute interval between GPS locations (assuming this has to do with transit versus foraging speeds typical for gannets?).

Added d’Entremont et al. 2022b as a reference. Tags recorded location every 15 minutes. The 30 minute interval was chosen to account for some GPS points failing to fix during foraging bouts (e.g. lack of fix due to being underwater repeatedly). Previously (while writing d’Entremont et al. 2022b), we found that the average travel speed of GPS points occurring before dives was 19.2 km/h with an SD of 17.6 km/h and that only 8% of these dive locations were based on the 30 min interval, with 92% within the 15 min interval. As such, any potential error in the 30 min dive locations was likely small and accounted for a small proportion of all recorded dive locations.

**Line 158**: No need to redo any analysis, but curious as to why latitude was not included as a covariate.

We added latitude to our model in the SD lat/lon spline as suggested by the previous reviewer.

**Line 161-163**: Please include information on what the splines were for each covariate (i.e. thin plate regression splines?). Out of interest, were any interaction terms explored for addition to the models, i.e. depth: SST or depth:slope?). Inclusion of interaction terms might help improve deviance explained especially of the V-shaped dive model?

We added the type of splines (penalized thin plate regression splines), and added that we tested for concurvity amongst the model components, namely the smooth terms.

**Line 165-170**: Please include a reference for the AUC and AIC methods.

We are now using a different method to select our final predictive model following Reviewer 1 advice.

**Line 188-189**: Are the statistical tests of this paragraph described in the methods? If test is among years then surely there should be at least 3 test statistics, i.e. 2019 vs 2020, 2019 vs 2021, 2020 vs 2021? Please also report degrees of freedom.

We used a Chi-squared test, which only has one test statistic indicating whether observed outcomes were different than expected.

See the following on L142: “We tested for the independence between year and dive shape using a Chi-square test to determine if there was an association in the distribution of dive shapes among years.”

Degrees of freedom have now been added in-text.

**Line 299**: Without going out and testing whether locations of hotspots of U shaped dives contain spawning capelin, or checking corresponding landings by gannets during the early chick-rearing period (i.e. similar to Montevecchi 2007), this is still conjecture, but it is a testable hypothesis for future studies.

We have added a reference regarding beach and deep-water capelin spawning sites in the region to help back up these claims.

See L335 where we have added the following: “Furthermore, areas deemed highly suitable for capelin foraging by gannets in our study overlaps with putative beach and deep-water capelin spawning areas identified by capelin fishers with extensive knowledge and experience of capelin distributions in the region (Bliss et al. 2023). “

**Line 343-344**: How do you know they are not going for cod during early chick-rearing?

It is plausible that a minute fraction of dives may have been directed towards cod. However, opportunistic diet samples collected from tagged birds, as well as samples collected from roosting gannets never contained cod (see d’Entremont KJN, Davoren GK, Walsh CJ, Wilhelm SI, Montevecchi WA (2022) Intra- and inter-annual shifts in foraging tactics by parental northern gannets Morus bassanus indicate changing prey fields. Mar Ecol Prog Ser 698:155-170. https://doi.org/10.3354/meps14164).

Also key to note, cod was a very rare dietary component in diet samples collected from gannets on nearby Funk Island from 1990-2020 (WAM unpubl. data).

**Line 362-364**: However, as gannets have been shown to have foraging plasticity, see for example Pettex et al. 2012., and are able to switch over the breeding season i.e. d’Entremont 2022b, it becomes a question of whether the gannets are able to compensate for changing capelin distributions…which is cause for continued monitoring of this colony and situation.

References
Pettex, Emeline, Svein Håkon Lorentsen, David Grémillet, Olivier Gimenez, Robert T. Barrett, Jean Baptiste Pons, Céline Le Bohec, and Francesco Bonadonna. 2012. “Multi-Scale Foraging Variability in Northern Gannet (Morus Bassanus) Fuels Potential Foraging Plasticity.” Marine Biology 159 (12): 2743–56. <https://doi.org/10.1007/s00227-012-2035-1>.

Included Pettex et al. 2012 as a reference and added the following the L412: “However, as gannets exhibit foraging plasticity within and between years (e.g. Pettex et al. 2012, d’Entremont et al. 2022b), they may be able to compensate for these distributional shifts by exploiting other prey species. Further monitoring of the foraging behaviour of parental gannets at Cape St. Mary’s is warranted to assess responses to these distributional shifts. “

**Reviewer #4**:

Title: Quantifying space-use of parental Northern Gannets (Morus bassanus) in pursuit of different prey types

This study aimed to assess the distribution and abundance of key forage fishes (namely 1 - capelin, and 2 – other pelagic forage species) in the western North Atlantic by investigating the foraging behaviour and space-use of northern gannets. The study deployed 25 GPS/Time-depth recorder devices on breeding Northern Gannets at Cape St. Mary’s, Newfoundland from 2019 to 2021. Gannets foraging in these waters have been shown to perform two distinctly shaped dives characteristic of the prey they are targeting. U-shaped dives are associated with capture attempts on capelin, while V-shaped dives are linked to other pelagic prey species. The authors used kernel density estimates and habitat suitability models to predict the spatiotemporal distribution of these two different dive types. They created three different habitat suitability models, one for U-shaped dives and two models for V-shaped dives, separated into early and late chick-rearing. The results of the study showed that space-use by gannets varied both within and between years, depending on environmental conditions and the prey they are exploiting. Both dive types generally occurred within shallow coastal waters and cool temperatures (11-15oC), although some V-shaped dives were also performed in waters of ~18oC, coincident with warmer prey species such as Atlantic saury. The authors suggest that regions defined as suitable for U-shaped dives are likely to represent consistent capelin spawning habitat. Given capelin’s importance for many predator species, the authors indicate that these areas, identified as important for spawning capelin, may also be of multi-species conservation concern.

I found this paper to be generally well written, the objectives clearly laid out and the methodology sound. I particularly enjoyed reading the approach for using dive types as a proxy for the distribution of different prey groups. This study makes a nice contribution to the toolkit of approaches for remotely monitoring the state of marine ecosystems. I have made largely minor comments throughout, which I hope the authors will find beneficial.

**General comments:

1.** This paper considers the distribution of U-shaped and V-shaped dives as a function of environmental conditions and largely ignores the issue of dietary preference. I would like to see some discussion around the effect of dietary preference in determining whether birds would be conducting proportionally more U or V-shaped dives. It seems to me that the study assumes that the preponderance of each dive (prey) type is driven entirely by their availability, without considering a preference for one prey type over another. So, if capelin were the preferred prey, would we not expect the preponderance of U-shaped dives to be nonlinearly related to its availability, remaining the dominant dive type until a threshold after which these dives would be rapidly replaced by V-shaped dives? I think this would be worth some thought, because if there is a level of preference, then clearly the probability of one dive type (V-shaped) occurring is dependent on the probability of the other (U-shaped) not occurring, no? And if there is a confounding effect of preference, this could have implications for how we use such methods to track changes in prey abundance and distribution.

Thank you for this interesting point! Owing to the phenology of capelin in this ecosystem, they do in fact become significantly less available in early August following their spawning period. In fact, mackerel are likely the “preferred” prey owing to mackerel being the most energetically dense prey option for gannets in the NW Atlantic (see Montevecchi et al. 1984). So if anything, I would think mackerel being available would influence gannets into performing more V-dives for this prey species. At that point, the response/shift to mackerel would be largely driven by their “preferred” prey now being available, as they migrate into the region in greater numbers. This shift away from capelin to mackerel, etc., is also coincident with increasing energetic demands of the chick, which has now been alluded to on L318.

**2.** The language shifts between present and past tense in a few places. Try to keep tenses consistent throughout.

We scanned and edited the document to keep tenses consistent.

**L. 62.** “as asymptomatic population growth”. This comes across as if the colony grew despite years of low productivity. I am not sure if this is what you intended, but either way I think this could be better worded.

Changed to “as well as plateaued population size”.

**L. 70.** remove “Clupea harengus” – already defined in L. 55.

Done.

**L. 71.** Change “Parental” to breeding, and remove the later “breeding”

Removed “Parental”.

**L. 71.** Either refer to Newfoundland in full throughout, or define NL with the first reference to Newfoundland.

Introduced “NL” as the abbreviation for Newfoundland on L48 and kept this consistent thereafter.

**L. 80.** …we aimed…

Done.

**L. 82**. Just refer to prey. To me, the reference to cold-blooded prey implies that they also eat warm-blooded prey, but that you didn’t consider these in this study.

Done.

**L. 82.** …for a better understanding…

Done.

**L. 102-103**. …Uria 300 GPS loggers with Temperature-Depth Recorders…

Done.

**L. 110.** Is the variance metric standard deviation?

Yes. Changed to “3547 g ± SD 459”.

**L. 112.** How do you define a diving bout?

Reworded to:

“…during dives (e.g. from first entry of water during dive until resurfacing).”

**L. 120.** It would be helpful to have a figure giving a representative example of a U- and V-shaped dive. This could either be in the main text or in the supplement.

U- and V- shaped dives by seabirds are well established in the literature, and as such we don’t believe it is necessary to reconstruct a figure displaying them. See Garthe et al. 2000 (which we have cited in-text) for an example. <https://doi.org/10.1098/rspb.2000.1200>

**L. 123.** This sentence is confusingly worded. Was a dive bout classified as the period of time over which the interval between consecutive dives remained less than 30 min?

See L129. Changed wording to the following: “ Foraging locations were considered to be any GPS location recorded within 30 minutes before a dive (e.g., d’Entremont et al. 2022b).

**L. 126-130.** This section could be better explained. It is not altogether clear to me why the authors chose to split V-shaped dives into an early and late season. I think the nature of the influence of U-shaped dives on V-shaped dives could be better described. Would it not be better to account for early/late season effects by fitting the models with a serial autocorrelation structure on date?

Please see above response to this same issue brought up by Reviewer 1. Refer to our response to Reviewer 1, regarding L142-143.

**L. 140-143.** “…we assume that the two dive profiles reflect prey type availability or preference”. I’m not sure I follow this. In my understanding, the distribution of dive shapes would differ considerably depending on whether they represented prey availability or prey preference. For example, if capelin is preferred, you might find the preponderance of U-shaped dives remains dominant even at low capelin densities and high relative availability of other prey. Whereas, if there is no preference, the dominance of dive types would be purely a function of relative availability of their different prey. I think it would be good to at least discuss how the findings here could be influenced by this.

Removed “or preference” from this sentence to focus on availability.

**L. 152.** This sentence may be better located earlier in the methods. i.e., when describing the fieldwork.

Deleted the sentence. We capture the study period fully in the “GPS Device Deployment” section. Beginning on L101.

**L. 155-160.** There should be a reference somewhere to the data products used for these analyses – specifically SST.

Added the following to L177: “SST data were retrieved from the Multi-scale Ultra-high Resolution SST Analysis fv04.1 (jplMURSST41) dataset on the Environmental Research Division’s Data Access Program (ERDDAP, https://coastwatch.pfeg.noaa.gov/erddap/index.html).”

**L. 159.** Were all variables extracted at their native resolution?

Yes.

**L. 160.** Were other oceanographic variables considered (e.g. chl a)?

Yes. Chl a was considered early on, but there was poor data coverage in our study area, leading to poor resolution. As such, we removed it from consideration for our models.

**L. 172.** It would be good to have reported the mean number of observations (dives) per day, given that predictions were conducted at a daily resolution.

Added the following information: “The mean number of dives per day across all three years was 70.8 ± 4.7 SE (n= 199), though the number of dives per day consistently decreased as the breeding season progressed in each year (Fig. 3).”

Also created a new figure for this.

**L. 185**. …predictions…

Fixed.

**L. 188.** “association” This could be more clearly stated. It’s not clear whether this implies that they were different or similar.

Changed to: “There was a significant temporal variation in the distributions of U- and V-shaped dives among years”.

**L. 231-232.** Remove “which included distance to the colony, longitude, bathymetry, slope, and sea surface temperature” – this is covered off by the previous sentence.

Done.

**L. 249-250.** Confusingly worded sentence

Changed wording to the following: “This response was different for late V-shaped dives, for which probability of occurrence in relation to depth was positive both for deeper (ca < 200 m) and shallower waters (ca > 100 m).”

**L 275-276.** I’m not sure I agree that an increased number of dives is indicative of decreased prey availability in the case of plunge diving species such as gannets. My understanding is that gannets locate prey from the air and thereafter initiate diving, whereas pursuit divers may undergo multiple exploratory dives to find prey (therefore having to increase dive frequency to locate prey when it is less abundant). Recent work has also found that the number of prey within a prey patch is not a predictor of foraging success (<https://doi.org/10.1111/1365-2656.12455>) – i.e. they don’t necessarily have to dive more frequently to have success on smaller prey patches. Therefore, I would have expected decreases in prey availability to be associated with concurrent decreases in the frequency of dives.

Added another reference with supporting evidence that gannets (Australasian in this case) increase foraging effort during times of poor prey availability: Angel LP, Barker S, Berlincourt M, Tew E, Warwick-Evans V, Arnould JPY. Eating locally: Australasian gannets increase their foraging effort in a restricted range. Biology open. 2015;4(10):1298–305. pmid:26369928

Added the following to L309: “Alternatively, it has been found in Cape Gannets that the number of prey in fish schools being exploited has no influence upon foraging success (Thiebault et al. 2015), albeit this study did not examine the influence of general prey abundance over a broader geographic range on dive effort (e.g., the number of schools within the foraging range of the gannets). Additionally, the notion that an increase in U-shaped dives was associated with lowered prey availability. This is consistent with the fact that capelin availability in Newfoundland waters was considered to be lower than usual in 2020 (d’Entremont et al. 2022b, Lescure 2021, G. Davoren pers. comm.).”

**L. 286.** “near” – perhaps towards is a better word choice?

Changed to “towards”.

**L. 294.** “This” – Together, these findings reinforce…

Changed wording of sentence to: “This further strengthens previous assertions that U-shaped dives by Northern Gannets in the western North Atlantic are primarily associated with foraging on spawning capelin (Garthe et al. 2000, Garthe et al. 2014, d’Entremont et al. 2022b).”

**L. 299.** “Areas deemed as highly” – Areas deemed highly…

Done.

**General discussion comments**

• There is considerable discussion around the loss of cold-water species (e.g. capelin) from the system and negative effects of this. I would be interested to see some speculation around a possible increased prevalence of warmer water species (e.g. saury), and their potential to (partially) compensate for the loss of colder species. I’m sure they wouldn’t be sufficient to maintain the population, but it might be an interesting discussion point.

Added the following to L428: “Such supplementation of alternative prey sources could be achieved by a potential increase in saury if average sea surface temperatures of ~18°C (the preferred temperature of Atlantic Saury, Dudley et al. 1985) are reached during the late chick-rearing period, although it remains to be seen how gannets would respond to such drastic shifts in the ecosystem. “

• I am wondering if you have access to fisheries data. Given that dive shapes are being linked to different prey types, I think it would be valuable to have the predicted distribution of dives (particularly U-shaped) plotted against the distribution of fishing activity/known spawning grounds. For example, a simple comparison could be to have a kernel density estimate of U-shaped dives overlaid by a kernel density estimate of capelin fishing effort, and/or polygons of their known spawning distribution.

Added the following to L335: “Furthermore, areas deemed highly suitable for capelin foraging by gannets in our study overlaps with putative beach and deep-water capelin spawning areas identified by capelin fishers with extensive knowledge and experience of capelin distributions in the region (Bliss et al. 2023). “ See reference here: <https://www.frontiersin.org/articles/10.3389/fmars.2022.1061689/full>

Unfortunately, this is the best we can do, as we do not have access to capelin fisheries data.

**Figures**

Fig. 5. It appears that the partial fits for bathymetry show an increase in dive probability over depths of <0 (i.e., over land). Is this just an artefact of inshore foraging close to coastal cliffs? In this case, it would be better to cap all bathymetry values >0 at 0.

We fixed the Bathymetry layer. The NOGA Domain polygon was not based on the GEBCO bathymetry layer, thus the land boundaries were not exactly aligning together. We fixed this by using the GEBCO bathymetry layer to define the land boundaries of the NOGA Domain polygon in which are generated the random locations at which depth values are extracted. Thanks for pointing this out to us.
