# Supplementary material for: Quantifying inter-annual variability on the space-use of parental Northern Gannets (Morus bassanus) in pursuit of different prey types
Source: PLoS One. 2023 Jul 14;18(7):e0288650. doi: 10.1371/journal.pone.0288650 (PMC10348513; doi:10.1371/journal.pone.0288650)
Supplement: Attachment — Submitted filename: Response to Reviewers_v2.docx [file pone.0288650.s004.docx]

Dear Dr. Paiva,

Please see below, our responses to the suggested edits made by the reviewers.

Many thanks,

d’Entremont et al.

Journal Requirements:

Please review your reference list to ensure that it is complete and correct. If you have cited papers that have been retracted, please include the rationale for doing so in the manuscript text, or remove these references and replace them with relevant current references. Any changes to the reference list should be mentioned in the rebuttal letter that accompanies your revised manuscript. If you need to cite a retracted article, indicate the article’s retracted status in the References list and also include a citation and full reference for the retraction notice.

Thiebault et al., 2015 has been removed at the request of Reviewer 4.

Wilhelm et al., 2015 has been removed from the References list as it is not cited in-text.

Reviewer #2: I want to congratulate the authors for the thorough revision they carried out. The manuscript is clear, with the statistical procedures and computations clearly stated and understandable. However, on reading through this revised version, I noticed a few typos or very minor grammar issues that I recommend the authors deal with before the acceptance.

I added a few comments and made my correction/suggestions in the file attached.

These minor comments/corrections have been accepted in the manuscript text.

Reviewer #3: I would like to congratulate the authors on a thorough and well-done revision. My comments have been beyond satisfactorily addressed. Congratulations on this interesting study!

Reviewer #4: I commend the authors on this very interesting study and I appreciate their effort to address the suggestions that I provided on the previous manuscript version. Overall, I am happy with their revisions and feel that they have sufficiently addressed my concerns. I have only a few very minor additional comments (largely typographical) for them to include prior to publication, which I have outline below. Note the Line numbers refer to the tracked changes document.

- L121. …(i.e. from first entry into water until resurfacing)…

Done.

- L.146. …early and late V-shaped dives.

Done.

- L.369-373. I appreciate that the authors have included this text in response to my previous comment. However, it appears to interrupt the flow and doesn’t really add anything – so maybe better to remove.

Sentence has been removed.

Perhaps the authors could instead consider changing the wording in L. 365. to: “Increased diving effort in pursuit-diving seabirds, including gannets…”

Changed to: “Increased foraging effort by seabirds, including gannets,…”

Also, my understanding is that, in the Angel et al. 2015 paper, the authors only found a significant difference in dive rate for male gannets and only in a single year. So, I’d suggest either changing “diving effort” to “foraging effort”, or removing the Angel et al. 2015 ref.
Changed to “foraging effort”.

- L427. …those sites…

Done.

- L475. …greater energy on foraging…

Done.

- L481. …results in an earlier spring bloom of primary producers…

Done.

- L537. …with foraging habitat suitability being heavily reliant on sea surface temperature thresholds of their poikilothermic prey.

Done.

- Methods – Habitat Suitability Modelling: I agree with why it wasn’t used, but I think it would still be good to mention why chl a was not included in the analyses – i.e. it was considered, but removed due to poor spatio-temporal coverage over the study period.

Added the following to L 178: “Chlorophyll a was also considered but removed from further models due to poor spatiotemporal coverage over the study period.”
